# Dual-Purpose Inoculants and Their Effects on Corn Silage

**DOI:** 10.3390/microorganisms8050765

**Published:** 2020-05-20

**Authors:** Dimas Hand Vidya Paradhipta, Seong Shin Lee, Byeongsam Kang, Young Ho Joo, Hyuk Jun Lee, Yeyeong Lee, Jinwoo Kim, Sam Churl Kim

**Affiliations:** 1 Division of Applied Life Science (BK21Plus, Insti. of Agri. & Life Sci.), Gyeongsang National University, Jinju 52828, Korea; dimazhand@gmail.com (D.H.V.P.); seongshin73@gmail.com (S.S.L.); forevertrue3@naver.com (B.K.); wn5886@gmail.com (Y.H.J.); hyukjun0209@gmail.com (H.J.L.); 2 Faculty of Animal Science, Universitas Gadjah Mada, Yogyakarta 55281, Indonesia; 3 Department of Plant Medicine, Gyeongsang National University, Jinju 52828, Korea; 2006123@naver.com (Y.L.); jinwoo@gnu.ac.kr (J.K.)

**Keywords:** antifungal, carboxylesterase, corn silage, lactic acid bacteria

## Abstract

This study was conducted to screen dual-purpose lactic acid bacteria (LAB) from uncontrolled farm-scale silage, and then we confirmed their effects on corn silage. The LAB were isolated from eight farm-scale corn silages, and then we screened the antifungal activity against *Fusarium graminearum* and the carboxylesterase activity using spectrophotometer with *p*-nitrophenyl octanoate as substrate and McIlvane solution as buffer. From a total of 25 isolates, 5M2 and 6M1 isolates were selected as silage inoculants because presented both activities of antifungal and carboxylesterase. According 16S rRNA gene sequencing method, 5M2 isolate had 100.0% similarity with *Lactobacillus brevis*, and 6M1 isolate had 99.7% similarity with *L. buchneri.* Corn forage was ensiled in bale silo (500 kg) for 72 d without inoculant (CON) or with mixture of selected isolates at 1:1 ratio (INO). The INO silage had higher nutrient digestibility in the rumen than CON silage. Acetate was higher and yeasts were lower in INO silage than in CON silage on the day of silo opening. In all days of aerobic exposure, yeasts were lower in INO silage than CON silage. The present study concluded that *Lactobacillus brevis* 5M2 and *L. buchneri* 6M1 confirmed antifungal and carboxylesterase activities on farm-scale corn silage.

## 1. Introduction

The contamination of undesirable microbes is one of the biggest concerns in the silage production. Growths of yeast and mold can degrade the nutrient content of silage and then produce the toxigenic compounds that are harmful for ruminant [1,2]. Moreover, those microbes can grow massively after silo open due to the presence of oxygen. In this situation, increasing populations of yeast and mold increase aerobic deterioration and decrease the shelf life of silage [3]. Previous studies reported that antifungal agents, such as propionic acid, sorbic acid, caproic acid, sodium benzoate, or potassium sorbate, could increase aerobic stability of silage [4,5]. Nevertheless, lactic acid bacteria (LAB) are also able to produce acetate, proteinaceous compounds, peptides, hydrogen peroxide, or reuterin that present the antifungal effect [6,7]. However, the ability of LAB to release antifungal substance is varied in each strain [7].

In the rumen, the percentage of digestible fraction from silage decrease with lignification of a plant cell. The rumen microbes are capable to digest cellulose or hemicellulose of the silage, but they could not produce the enzymes to breakdown lignin complex [8]. The hydrolysis of lignin complex in the rumen might occur by the presence of carboxylesterase. Application of carboxylesterase, such as ferulate esterase, in the silage was proved to increase the degradation of fiber in the rumen [9,10]. Recently, some of the studies reported that several strains of LAB had the ability to produce carboxylesterase, even though its improvement on nutrient digestibility was inconsistent results depending on bacteria strains and forage sources [11,12,13]. 

The effectiveness of silage inoculants might be different depending on isolation sources. Most of the silage inoculants were isolated from fermented foods [7,14,15], but limited inoculants were isolated from silage. Similar to food fermentation, not all LAB strains can be used as silage inoculant [15,16]. Specific strains of LAB that survive in the ensiling condition and drop the pH rapidly are required for making silage [15,16]. Screening inoculant from uncontrolled fermentation, such as farm-scale silages, can provide more various isolates that may be more appropriate to adapt with the actual ensiling condition in the silage. In the viewpoint of field application, the purpose of the present study was to find an appropriate dual-purpose inoculant that could adapt and improve silage quality in the farm-scale condition. In the present study, LAB were isolated from uncontrolled farm-scale corn silages and screened based on their antifungal activity to inhibit *Fusarium* growth and their carboxylesterase production. Two selected strains were used as silage inoculant to investigate their effects on the rumen digestibility, fermentation characteristics, and aerobic stability of farm-scale corn silage. 

## 2. Methods and Materials

### 2.1. Corn Silage Collection as Isolate Source

Uncontrolled corn silages (*n* = 8) were obtained randomly from eight beef cattle farms in Sancheong, Gyeongnam province, Korea, as isolate sources for LAB. The ensiling procedures of these corn silages including hybrid, inoculant application, and storage time were unknown. One kilogram of corn silage was collected representatively from bale silo (about 500 kg) in each farm. The collected corn silage was sub-sampled 20 g to make silage extraction and 5 g to isolate LAB. Silage extraction was made by blending 20 g silage with 200 mL of sterile ultrapure water for 30 sec and then filtered through two layers of cheesecloth [17]. The silage extraction was used to evaluate fermentation characteristics of corn silage consisting of pH, ammonia-N, lactate, and volatile fatty acid (VFA) and microbial counts, including LAB, yeasts, and molds. In addition, Flieg’s score was calculated based on the lactate and VFA (i.e., acetate, propionate, and butyrate) concentrations of silage described by Flieg [18]. Protocols to analyze fermentation characteristics and microbial counts are described in the next section, Materials and Methods.

### 2.2. Lactic Acid Bacteria Isolation

Collected corn silage was mixed with two different liquids, consisting of sterile distilled water or milk to harvest isolates. In distilled water, silage was incubated with ratio 1:100 and shaken at 220 rpm for 1 h to make silage suspension. In milk, silage was incubated with ratio 1:50 and incubated at 28 °C for 48 h to make curd. Then, either silage suspension or curd was serially diluted (10^−3^–10^−8^) with sterile distilled water. Aliquots of 100 µL of each dilution were spread on de Man, Rogosa, and Sharpe (MRS; Difco, Detroit, MI, USA) agar medium. The medium was incubated at 28 °C for 5 d in a CO_2_ incubator (Thermo Scientific, Waltham, MA, USA). All isolates were tested for the antifungal activity against *Fusarium graminearum* and the carboxylesterase activity. Two LAB strains with the highest antifungal and carboxylesterase activities were selected, mixed, and used as silage inoculant.

### 2.3. Antifungal and Carboxylesterase Activities

All isolates were performed against *Fusarium graminearum* head blight fungus MHGNU F132 to determine antifungal activity. The *F. graminearum* head blight fungus MHGNU F132 was isolated from the diseased corn plant. Hyphal colony of *F. graminearum* was grown on potato dextrose agar (PDA; Difco, Detroit, MI, USA), and it was placed on the center of plate. Each bacterial isolate was streaked 2 cm from the center in triplicate. *F. graminearum* grown in the plate without a streaked isolate was used as a negative control. All the PDA plates were incubated at 28 °C for 8 d in an aerobic incubator (Johnsam Corp., Boocheon, Korea), and then fungal growth inhibition was measured by calculating the growth area of *F. graminearum* from the center to the bacterial isolate along 2 cm. The inhibition area was expressed in cm unit.

Carboxylesterase activity assay from each isolate was conducted according to the protocol described by Pérez-Martín et al. [19], with some modifications. Each isolate was cultured anaerobically in MRS at 28 °C for 5 d. Each isolate was measured the carboxylesterase activity in triplicate. Bacterial cells were harvested by centrifugation at 7558*× g* for 1 min and washed twice with sterile normal saline. Then, cell population was adjusted at an OD_600_ 0.5 with sterile normal saline. The *p*-nitrophenyl octanoate (Sigma-Aldrich, Madrid, Spain) was diluted with ethanol to make 25 mM stock solution. Carboxylesterase activity assay was carried out by making a total 1 mL reaction solution; 860 µL 0.1 M McIlvane buffer (0.1 M citric acid and 0.2 M K_2_HPO_4_), pH 5.0, 100 µL each bacterial suspension, and 40 µL substrate stock solution (1 m*M* final substrate concentration) [20]. Reactions were incubated at 37 °C for 2 h, then 100 µL of 0.5 *M* NaOH was added to stop the reaction, and the absorbance was measured at 320 nm using a spectrophotometer (GENESYS 10S UV-Vis, Thermo Scientific). The values were calculated using the Miller unit (MU) [21]. 

### 2.4. Identification Using the 16S rRNA Gene Sequencing 

The partial 16S rRNA gene region was amplified using polymerase chain reaction (PCR); Premix (Bioneer, Seoul, South Korea) and universal primers 27F (5-AGAGTTTGATCMTGGCTCAG-3) and 1492R (5-GGYTACCTTGTTACGACTT-3). PCR reaction was performed by initial denaturation at 98 °C for 4 min, followed by 30 cycles denaturation at 98 °C for 30 sec, annealing at 55 °C for 30 sec, and elongation at 70 °C for 1 min 30 sec, followed by the final extension step of 72 °C for 4 min. PCR product confirmed using 0.8% agarose gel was purified using the PCR purification kit (Expin PCR SV, GeneAll). Sequencing was performed by Macrogen sequencing service (Macrogen, Daejeon, South Korea).

Sequences were analyzed using the BLAST program (http://www.ncbi.nlm.nih.gov/blast/) [22]. Phylogenetic analysis was performed using the neighbor-joining method and Tajima-Nei distance modeling in MEGA 7.0 [23]. The sequences of related *Lactobacillus* spp. were downloaded from GenBank.

### 2.5. Inoculant Preparation

Previously, two screened LAB (*Lactobacillus brevis* 5M2 and *L. buchneri* 6M1) from agar plate were grown, respectively, in lactobacilli MRS media and then incubated at 28 °C for 5 d in a CO_2_ incubator. After incubation, these LAB were adjusted with sterile ultra-pure distilled water to obtain 1 × 10^5^ colony forming unit (cfu)/g of fresh forage as the recommended application rate. Furthermore, two screened LAB were mixed at ratio 1:1 for silage inoculant. 

### 2.6. Silage Production

Corn forage (Kwangpyeongok hybrid) at ½ milk line stage was harvested from 20 ha of the cornfield at Sancheong Agricultural Research and Extension Service (Sancheong, Gyeongnam province, South Korea) and chopped at 3–5 cm length using a Class of Jaguar 850 forage harvester (Class of America, Columbus, IN, USA). About 500 kg of chopped corn forage was ensiled for 72 d into the bale silo and treated with 1% of sterile distilled water in fresh forage (CON) or 1% of inoculant (1 × 10^5^ cfu/g) in fresh forage (INO). Each treatment consisted of 4 silos as replications. Both the sterile distilled water and the silage inoculant were sprayed individually for each bale silo with a manual sprayer when forage was being rolled in Round Baler (MR-820, Takakita Co., Ltd., Natsumi, Japan), and then the baled forage was wrapped using Automatic Bale Wrapper (WM1120, Takakita Co., Ltd., Natsumi, Japan). All bale silages were stored into the barn to protect from animal and weather damage. The fresh corn forage collected from the bale chamber and the silage after ensiling were sub-sampled, respectively, at 2 kg for chemical composition and in vitro digestibility analyses. Furthermore, silage was sub-sampled at approximately 20 g to make the silage extraction. The aerobic stability was measured by sub-sampled 2 kg of silage and placed in aerobic condition. 

### 2.7. Chemical Compositions and In Vitro Rumen Digestibility

The sub-sampled forage and the silage were dried at 65 °C for 48 h and ground to pass 1-mm screen using a cutting mill (Shinmyung Electric Co., Ltd, Gimpo, Korea) for the measurement of chemical compositions and in vitro digestibility for 48 h. Dry matter (DM) was determined by drying 10 g of sample into an forced-draft oven (OF-22GW, Jeio Tech, Seoul, South Korea) at 105°C for 24 h. Crude ash (CA) was determined with a muffle furnace at 550 °C for 5 h. Crude protein (CP) and ether extract (EE) were determined by the producers of Kjeldahl (method 984.13 of AOAC) using N analyzer (B-324, 412, 435, and 719 S Titrino, BUCHI, Flawil, Switzerland) and Soxhlet (method 920.39), respectively [24]. The neutral detergent fiber (NDF; method 2002.04) and acid detergent fiber (ADF; method 973.18) were determined by using an Ankom^200^ Fiber Analyzer (Ankom Technology, Macedon, NY, USA) [24]. The NDF was analyzed with heat stable amylase and expressed inclusive of residual ash, while the ADF was also expressed inclusive of residual ash. Hemicellulose (HEMI) was determined by calculating the differences between NDF and ADF. 

For in vitro digestibility, the procedure of animal care for cannulated cows was approved by Animal Ethical Committee of Gyeongsang National University, Jinju, South Korea. Rumen fluid (pH 6.68) was collected from two non-pregnant cannulated Hanwoo heifers before morning feeding. Their diets consisted of tall fescue hay and commercial concentrate mixture at 8:2 ratio, plus vitamin-mineral premix. Three replications for each silo along with three blanks were designed for analysis of in vitro digestibility. The in vitro DM digestibility (IVDMD) was determined after incubating a dried sample (0.5 gram) with rumen buffer for 48 h described by Tilley and Terry [25] using an Ankom Daisy^II^ (Ankom Technology, Macedon, NY, USA). The in vitro NDF digestibility (IVNDFD) was estimated after the analysis of dried samples and residues from the IVDMD assay for NDF. Then, the IVDMD and the IVNDFD were calculated as a percentage of DM (g/kg DM).

### 2.8. Fermentation Characteristics and Microbial Counts

Silage pH and ammonia-N were measured using pH meter (SevenEasy, Mettler Toledo, Greifensee, Switzerland) and the colorimetric method described by Chaney and Marbach [26], respectively. The silage extraction was centrifuged at 5645× *g* for 15 min to separate the supernatant and the silage residue. The supernatant was used for lactate and VFA analyses. The concentrations of lactate and VFA were determined using HPLC (L-2200, Hitachi, Tokyo, Japan) fitted with a UV detector (L-2400; Hitachi, Tokyo, Japan) and a column (Metacarb 87H; Varian, Palo Alto, CA, USA) according to the method described by Muck and Dickerson [27]. 

Silage extract (first dilution) was continued in several dilutions (10^−3^–10^−7^) to determine microbial counts, such as LAB, yeasts, and molds. The silage extract was plated in triplicate selective agar medium. The lactobacilli MRS agar media was used for LAB count, and the PDA for counting yeasts and molds. The MRS agar plates were placed in a CO_2_ incubator at 28 °C for 48 h, while PDA plates were incubated at 28 °C for 72 h in an aerobic incubator. Visible colonies were counted from the plates and the number of cfu was expressed per gram of silage. The microbial data was transformed to log10.

### 2.9. Aerobic Stability

After 72 d of ensiling, one kilogram of sub-sampled silage was located in a polystyrene box with aerobic condition and stored at room temperature (10 ± 1 °C) for 7 d. The temperature was recorded by a thermocouple, a sensor (MORGAN TR-60CH, Hong Kong, China) that placed at the geometric center of each sample. Two sensors as a laboratory replicates were used for each silo with three sensors to record the room temperature. Data were collected every 30 min. Aerobic stability was measured by the time (h) before a 2 °C increase in silage temperature above the ambient temperature [17]. The other 1 kg of sub-sampled silage was also located in the same aerobic condition with the same container type to measure changes of silage pH and microbial count, such as LAB, yeasts, and molds. For these analyses, 50 g of silages were sub-sampled on 1, 2, 3, 4, 5, and 7 d of aerobic exposure. The analysis procedures of pH and microbial counts were the same as described in the previous section.

### 2.10. Statistical Analysis

A total of 25 isolates was obtained and tested the activities of antifungal and carboxyl esterase. The statistical analysis considered a standard error value to declare the differences of antifungal and carboxylesterase activities between isolates. To measure the effect of selected isolate in the silage, two treatments (CON vs. INO) were applied in corn silage with 4 replications, respectively. Furthermore, all silage data in the present study were analyzed using General Linear Model of Statistical Analysis System 9.3 (PROC GLM; SAS, Version 9. Cary, NC, USA) [28]. The statistical model was *Y_ij_ = µ + T_i_ + e_ij_,* where *Y_ij_* = response variable, *µ* = overall mean, *T_i_* = the effect of inoculant *i*, and *e_ij_* = error term. Mean separation was performed by Student’s t-test to declare the significant difference at *p* ≤ 0.05 between two treatments. In addition, the polynomial contrasts analysis also used PROC GLM to evaluate the effects of increasing aerobic day (linear, quadratic, or cubic) on silage pH and microbial counts, including LAB, yeasts, and molds. Orthogonal coefficients for linear, quadratic, and cubic contrast were adjusted to account for the unequal spacing of observation day for aerobic exposure (0, 1, 2, 3, 4, 5, and 7 d) with Interactive Matrix Programming Language procedure of SAS (PROC IML) before testing polynomial contrast for the significance at *p* ≤ 0.05 [28].

## 3. Results

### 3.1. General Information of Collected Corn Silages as Isolate Source

The mean concentrations of DM, lactate, and acetate in corn silages collected from the beef cattle farms were 292, 51.5, and 8.93 g/kg, respectively (Table 1). The means of silage pH and Flieg’s score from all collected silage were 3.90 and 96.2. The propionate and butyrate of corn silages were not detected. The mean counts of LAB and yeasts were 6.66 and 6.12 log10 cfu/g, respectively. Molds were only detected from farm2, farm5, and farm8, where its mean count was 5.00 log10 cfu/g.

### 3.2. Antifungal and Carboxylesterase Activities of Isolates

**Figure 1:**
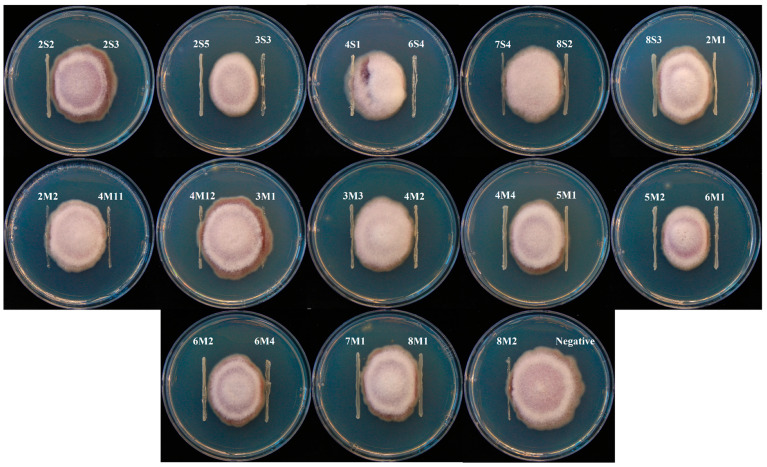
Plate assays of lactic acid bacteria (LAB) isolates against *F. graminearum* after 8 d of incubation.

**Figure 2:**
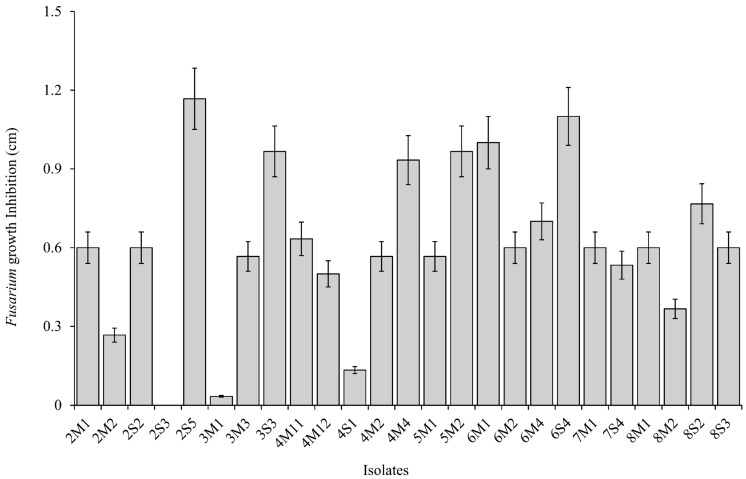
The inhibition area of *F. graminearum* by LAB isolates to measure antifungal activity. Error bar indicated standard error.

**Figure 3:**
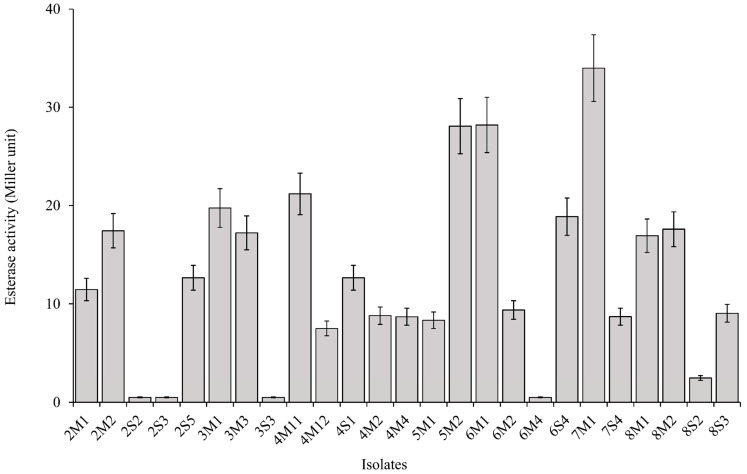
Carboxylesterase activity of LAB isolates based on Miller protocol. Error bar indicated standard error.

A total of 25 LAB isolates were collected from eight corn silages. The mean value of inhibition area of *Fusarium* growth from all isolates was 0.61 cm. Six isolates, consisting of 2S5, 3S3, 4M4, 5M2, 6M1, and 6S4, were classified as high antifungal activity because they presented a relatively higher inhibition area of *Fusarium* growth than the mean value, as well as the other isolates (Figure 1 and Figure 2). The inhibition areas of *Fusarium* growth from these isolates were 1.17 ± 0.12, 0.97 ± 0.10, 0.93 ± 0.09, 0.97 ± 0.10, 1.00 ± 0.10, and 1.10 ± 0.11 cm, respectively. On the other hand, the mean value of carboxylesterase activity from all isolates was 12.8 MU. Three isolates, consisting of 5M2, 6M1, and 7M1, could be classified as high carboxylesterase activity, which presented a carboxylesterase activity at 28.0 ± 2.80, 28.2 ± 2.82, and 33.9 ± 3.39 MU, respectively. These isolates showed a relatively higher carboxylesterase activity than the mean value, as well as the other isolates (Figure 3). In general, the highest antifungal activity was shown in the 2S5 isolate, and the highest carboxylesterase activity was shown in the 7M1 isolate. However, 5M2 and 6M1 isolates exhibited high in both activities of antifungal and carboxylesterase, which were subjected to further silage inoculants in the present study.

### 3.3. Identification of Isolates Using the 16S rRNA Gene Sequencing

**Figure 4:**
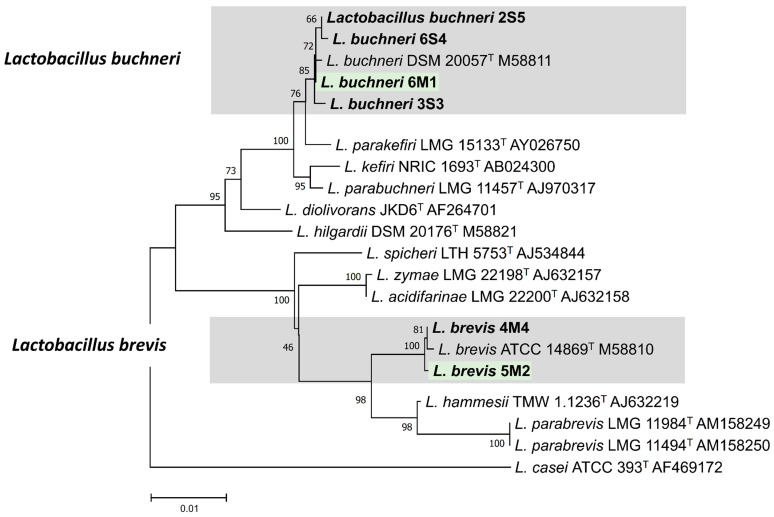
Phylogenic trees of the 16S rRNA gene sequences of 2S5, 3S3, 4M4, 5M2, 6S4, and 6M1 isolates.

Analysis of nucleotide sequences presented that isolates of 2S5, 3S3, 6M1, and 6S4 shared more than 99.6% similarity with *L buchneri*, while isolates of 4M4 and 5M2 shared 100% similarity with *L. brevis* (Table 2). In the phylogeny, 2S5, 3S3, 6M1, and 6S4 clustered in the same group as *L. bruchneri*, while 4M4 and 5M2 clustered in the same group as *L. brevis*, respectively (Figure 4). Based on the 16S rRNA gene region sequencing, 2S5, 3S3, 6M1, and 6S4 were identified as *L. buchneri*, while 4M4 and 5M2 were identified as *L. brevis*.

### 3.4. Chemical Compositions and In Vitro Digestibility of Forage and Silage

The mean concentrations of DM, CP, NDF, ADF, HEMI, IVDMD, and IVNDFD of corn forages before ensiling were 284, 86.7, 470, 213, 665, and 451 g/kg, respectively (Table 3). After 72 d of ensiling, INO silage had lower concentrations of NDF (*p* = 0.013; 456 vs. 493 g/kg) and ADF (*p* = 0.006; 250 vs. 275 g/kg) with higher IVDMD (*p* = 0.029; 652 vs. 625 g/kg) and IVNDFD (*p* < 0.001; 484 vs. 444 g/kg) than those of CON silage (Table 4). The other chemical compositions were not affected by inoculant application.

### 3.5. Fermentation Characteristics and Microbial Counts of Silage

The INO silage had higher acetate concentration (*p* = 0.019; 24.9 vs. 20.9 g/kg) and lower lactate to acetate ratio (*p* = 0.002; 5.22 vs. 6.22) than those of CON silage (Table 5). The silage pH and concentrations of ammonia-N and lactate were not affected by inoculant application. Propionate and butyrate were not detected in the present study. The yeast count (*p* = 0.029; 5.79 vs 5.89 log10 cfu/g) was lower in INO silage than in CON silage, while LAB count was not affected by inoculant application. Molds were not detected in the present study.

### 3.6. Aerobic Stability of Silage

**Figure 5:**
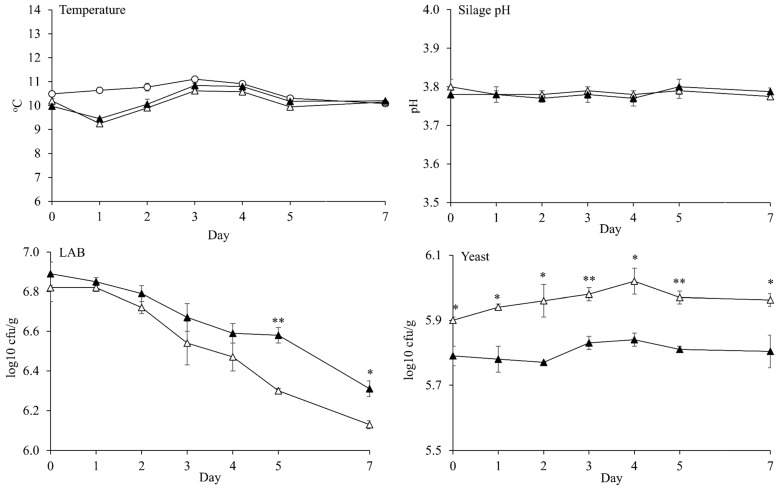
Temperature, silage pH, lactic acid bacteria (LAB) count, and yeast count of corn silage over 7 d of aerobic exposure. Room temperature (O); and Corn silage without inoculant application at ensiling (△) or with inoculant application at ensiling (*L. brevis* 5M2 and *L. buchneri* 6M1 at 1:1 ratio) (▲). Values differ between groups within same day *: *p* ≤ 0.05 and **: *p* ≤ 0.01. Based on polynomial contrast analysis, LAB count (*p* < 0.001) linearly decreased, while the pH (*p* = 0.873) and yeast count (*p* = 0.217) were in a stable pattern. The temperatures of room and silage were not a statistical analyzed. The molds were not detected in the present study. Error bar indicated standard error.

The silage temperatures were lower or similar with room temperature over 7 d of aerobic exposure. Thus, the aerobic stability of silage could not be determined in the present study. There was no significant different between CON silage and INO silage on silage pH over 7 d of aerobic exposure (Figure 5). The LAB counts of both silages decreased linearly (*p* < 0.001) by the day of aerobic exposure, whereas silage pHs (*P* = 0.873) and yeast counts (*p* = 0.217) were stable. The INO silage had a higher LAB count than CON silage at 5 d (*p* = 0.010; 6.58 vs. 6.30 log10 cfu/g) and 7 d (*p* = 0.020; 6.31 vs. 6.13 log10 cfu/g) of aerobic exposure. However, INO silage had a lower (*p* < 0.05) yeast count than CON silage over 7 d of aerobic exposure. Molds were not detected over 7 d of aerobic exposure in the present study (data was not presented).

## 4. Discussion

### 4.1. Screening Isolate

Collected corn silages from eight different farms showed various DM content, pH, and VFA profiles. This means that these corn silages had a different environmental growth for LAB, which might provide more variation of isolate strains for isolation procedure. All collected corn silages, except from farm8, had normal ranges of pH (3.7–4.0), ammonia-N (> 1 g/kg DM), lactate (30–60 g/kg DM), and classified as ‘very good silage’ based on Flieg’s score [4,29]. Even though classified as very good silage, the presence of acetate was in low concentration in all silages (< 20 g/kg DM). In fact, acetate is one of the antifungal compounds that can inhibit growths of yeast and mold and then increase aerobic stability of silage [6]. In addition, molds were still detected in the silages from farm2 and farm5. Besides being due to low acetate concentration, detected mold in these corn silages could be caused by their infection before ensiling [1,2,30]. As high energy forage, corn forage has a high potential of being overgrown by undesirable microbes before harvested [1,2]. According to Scudamore and Livesery [30], mycotoxin mold, such as *Fusarium,* are aerobically infected and associated with corn in the field. In addition, damp weather and drought stress of forage become factors that accelerate the increase of mold infection in the field [30]. The investigation of corn silage quality from beef cattle farm in the present study indicated that application of antifungal-producing inoculant was strongly recommended to inhibit the growth of undesirable microbes in corn silage, although the ensiling process conducted in good condition. Other than that, most corn silage in South Korea is formulated with other grains to make a total mixed ration, in which the presence of antifungal-producing inoculant may have beneficial effects against the *Fusarium* or the other molds in the diet. 

According to Alonso et al. [1], the major species of mycotoxin mold that contaminate forage or feedstuff could be varied depending on the area and climate. The *F. graminearum* was chosen to test the antifungal activity of isolates in the present study because it is the major mycotoxin mold of corn plant in South Korea [31]. Moreover, the *F. graminearum* could be still detected in the corn silage after ensiling that could produce mycotoxins and potentially decreased the aerobic stability of silage [1,2,3]. On the other hand, many previous studies mainly focused to screen the LAB that produced specific carboxylesterase, such as ferulic acid esterase [10,11,13]. However, all isolates were screened for general types of carboxylesterase in the present study. It was conducted because of that consideration that there is a large number of carboxylesterase besides ferulic acid esterase, such as acetyl xylan esterase, p-coumaric esterase, and glucuronyl methylesterase, that can breakdown the ester linkages between lignin and hemicellulose and then enhance the fiber degradation in rumen [9,32]. From a total of 25 isolates, there were several potential isolates that could be the candidate for silage inoculant. Six isolates, consisting of 2S5, 3S3, 4M4, 5M2, 6M1, and 6S4, inhibited *Fusarium* growth more than 0.90 cm, whereas the average inhibition area from all isolates was only 0.61 cm. Besides that, three isolates, consisting of 5M2, 6M1, and 7M1, presented carboxylesterase activity more than 25 MU, whereas the average carboxylesterase activity from all isolates was only 12.8 MU. In the present study, both 5M2 and 6M1 were classified as high antifungal and carboxylesterase activities. Thus, 5M2 and 6M1 isolates was selected and would be applied in the corn silage to confirm their activities. Identification isolates was conducted based on phylogenic trees of the 16S rRNA gene sequences, of which 6M1 isolate was identified as *L. buchneri*, while 5M2 isolate was identified as *L. brevis*. 

### 4.2. Applying Screened Isolates on Corn Silage

The chemical compositions of corn forage used in the present study were in normal range for corn grown in South Korea [33]. After ensiling, the concentrations of DM, CP, and EE were not affected by inoculant application in the present study. It could have occurred because the low pH in all silages (Table 5) was acid enough to inhibit the nutrient degradation during ensiling [4]. On the other hand, lower NDF and ADF concentrations in INO silage than in CON silage could be caused by carboxylesterase activity that was produced by *L. brevis* 5M2 and *L. buchneri* 6M1. The application of esterase-producing inoculant in the present study showed similar results as Lynch et al. [14], who reported the decrease of NDF concentration in alfalfa silage by exogenous fibrinolytic enzymes. Besides the inoculant, other factors also could influence the degradation of fiber compound in the silage during ensiling, such as a natural plant enzyme or acid hydrolysis [4,34]. The IVDMD was 27 g/kg higher in inoculated silage, while IVNDFD showed improvement at 4.4 g/kg. Caboxylesterase by *L. brevis* 5M2 and *L. buchneri* 6M1 was effective in solubilizing the lignin complex of corn silage in the present study and increased the accessibility of other fibirinolytic enzymes by rumen microbes to degrade cellulose or hemicellulose [9,10,32]. The results of IVDMD and IVNDFD in the present study supported the results of other previous studies [11,13]. In the viewpoint of animal performance, the increase of rumen digestibility, such as the result of the present study, could potentially have had improvement on feed efficiency, average daily gain, or milk production of the ruminant [9,35]

In the present study, pHs of INO silage and CON silage were lower than 4.0, which indicated a good acidification to inhibit the degradation of nutrient by natural biochemical reaction or undesirable microbes [4,29]. A good acidification could be proven by low ammonia-N concentration (> 1 g/kg DM) and no presence of butyrate and mold in the both of silages. The high lactate concentration in both INO silage and CON silage caused rapid acidification to decrease pH lower than 4.0 in both silages [4,29]. As high energy forage, corn silage can produce high lactate and low ammonia-N without application of an inoculant [29]. This was a reason there was no effect of lactate and ammonia-N by inoculant application in the present study. Selected isolates in the present study confirmed an antifungal activity in corn silage by high acetate production in INO silage. Acetate is known as an antifungal substance [3,6], which has been proven to inhibit yeast growth in INO silage (Table 5). In addition, our selected inoculants are classified as hetero fermentative LAB, which are able to convert lactate into acetate during ensiling [4,36]. 

On the other hand, LAB count on the day of silo opening was not affected by inoculant application in the present study. Even though it could improve fermentation characteristics, inoculation of LAB in the silage did not always promise to increase its count after silo opening. It was reported by previous studies that showed similar LAB count between inoculated silage and control silage [11,37]. In the present study, increase of acetate concentration by inoculant application reduced yeast count in INO silage on the day of silo opening, which supported the results of previous studies [5,6,13,37]. Moreover, the presence of acetate in silage could reduce aerobic deterioration after silo opening [3,6]. Molds were not detected in all silages on the day of silo opening because low pH in all silages were enough acid to inhibit its growth in the present study [5,33].

Aerobic stability of silages in the present study could not be determined due to the low temperature of environment during observation. There was no increase of silage temperatures more than 2 °C over 7 d of aerobic exposure. In addition, Lee et al. [33] explained that the increase of silage temperature during aerobic exposure is associated with mold and yeast growths. The mean room temperature in farm storage for 7 d of aerobic exposure was 10.7 °C, which inhibited yeasts or molds to grow optimally and to degrade the nutrient content in the silage [4]. The low room temperature during observation in the present study occurred because the bale silo was opened in early December, which followed the farming practice in the field and prevented the silage from freezing. At normal room temperature (20–25 °C), aerobic stability of corn silage with the same hybrid as in the present study was about 25.5–32.8 h [33], while, in the other study, the general aerobic stability of corn silage with various hybrids (Pioneer 31Y43, Pioneer 32D99, Croplan Genetics 827, and Croplan Genetics 799) was about 24.1–25.1 h [17]. Compared to normal temperature, the aerobic stability could be higher at low temperature because growths of yeast and mold were inhibited.

The silage pH was in a stable pattern during aerobic exposure, which supported the results of the stable yeast growth and the mold absence in the present study. No change of silage pH in the present study indicated less degradation of silage nutrient and low production of metabolite product by aerobic bacteria [3,4]. The LAB count linearly decreased during observation because LAB could not live optimally in aerobic condition [4]. Even though the growth of LAB linearly decreased, INO silage had higher LAB count than CON silage after 5 d of aerobic exposure. This result could indicate a higher aerobic tolerance of our selected isolates in INO silage than epiphytic LAB in CON silage. The yeast growth in all silages was stable, and there was no presence of mold during aerobic exposure in the present study due to low temperature. Paradhipta et al. [37] also reported a similar result as the present study where yeasts were grown slowly (7.61 to 7.94 log10 cfu/g), and no mold were detected on sorghum-sudangrass silage over 8 d of aerobic exposure at low room temperature (10 °C). Nevertheless, the beneficial effects of inoculant application in the present study can be found by lower yeast count in INO silage than in CON silage for 7 d of aerobic exposure, which was caused by the presence of acetate. 

## 5. Conclusions

Based on the screening result, *L. brevis* 5M2 and *L. buchneri* 6M2 presented high both activities of antifungal and carboxylesterase in the present study. The mixture of both new inoculants showed the improvement on corn silage quality by increasing nutrient digestibility and reducing contamination of yeast. Even though the aerobic stability of silage could not be defined, the lower yeast count and higher LAB count by new inoculant application indicate the beneficial results of antifungal activity. Therefore, this study concluded that *L. brevis* 5M2 and *L. buchneri* 6M1 confirmed antifungal and carboxylesterase activities on farm-scale corn silage.

## Figures and Tables

**Table 1:** Dry matter content, fermentation characteristics, and microbial counts of corn silages collected from beef cattle farms as isolate sources (n = 8).

Item	Source							
	Farm1	Farm2	Farm3	Farm4	Farm5	Farm6	Farm7	Farm8
Dry matter, g/kg	230	328	370	264	363	281	244	256
Fermentation characteristics								
pH	3.67	4.09	3.94	3.65	3.89	3.68	3.74	4.55
Ammonia-N, g/kg DM	0.92	0.64	0.80	0.45	0.70	0.53	0.56	1.29
Lactate, g/kg DM	96.1	29.6	55.8	48.0	51.7	62.5	50.6	17.5
Acetate, g/kg DM	16.7	8.08	4.57	10.8	5.51	4.82	8.98	12.0
Propionate, g/kg DM	ND^2^	ND	ND	ND	ND	ND	ND	1.21
Butyrate, g/kg DM	ND	ND	ND	ND	ND	ND	ND	ND
Flieg’s Score ^a^	100	94	100	98	100	100	100	78
Microbial count, log10 cfu/g								
Lactic acid bacteria	6.59	7.22	6.15	6.30	7.15	6.00	7.45	6.40
Yeast	6.00	7.13	5.70	5.65	6.39	5.30	6.57	6.18
Mold	ND	5.00	ND	ND	5.00	ND	ND	5.00

^a^: 0–20, failure; 21–40, poor; 41–60, satisfactory; 61–80, good; 81–100, very good.

**Table 2:** The identifications of LAB isolates using 16S rRNA gene sequencing.

Isolates	Nucleotide (bp)	Species	Identity (%)
2S5	1466	*L. buchneri*	99.86
3S3	1438	*L. buchneri*	99.93
4M4	1449	*L. brevis*	100.0
5M2	1454	*L. brevis*	100.0
6M1	1446	*L. buchneri*	99.86
6S4	1446	*L. buchneri*	99.65

**Table 3:** Chemical compositions of corn forages before ensiling (g/kg dry matter (DM)).

Item ^2^	Treatment ^1^		SEM	*P*-Value
	CON	INO		
**Dry matter**	**283**	285	4.37	0.689
Crude protein	86.4	87.0	6.04	0.897
Ether extract	33.8	34.8	0.98	0.240
Crude ash	47.4	45.9	2.00	0.401
Neutral detergent fiber	470	469	6.52	0.860
Acid detergent fiber	212	213	7.47	0.798
Hemicellulose	259	256	4.62	0.471
IVDMD	668	662	29.7	0.758
IVNDFD	449	452	38.3	0.936

^1^: CON, corn silage without inoculant application; INO, corn silage inoculated with mixture of *L. brevis* 5M2 and *L. buchneri* 6M1 at ratio 1:1. ^2^: IVDMD, in vitro dry matter digestibility; IVNDFD, in vitro neutral detergent fiber digestibility.

**Table 4:** Effects of new inoculant on chemical composition of corn silage ensiled for 72 d (g/kg DM).

Item ^2^	Treatment ^1^		SEM	*P*-Value
	CON	INO		
**Dry matter**	**266**	268	2.31	0.259
Crude protein	89.8	89.6	1.60	0.833
Ether extract	42.7	42.3	3.49	0.907
Crude ash	46.9	45.8	1.25	0.193
Neutral detergent fiber	493 ^a^	456 ^b^	14.8	0.013
Acid detergent fiber	275 ^a^	250 ^b^	8.59	0.006
Hemicellulose	218	207	8.36	0.100
IVDMD	625 ^b^	652 ^a^	11.7	0.029
IVNDFD	444 ^b^	484 ^a^	4.08	<0.001

^a,b^: Mean in the same row with different superscripts differ significantly (*p* ≤ 0.05). ^1^: CON, corn silage without inoculant application; INO, corn silage inoculated with mixture of *L. brevis* 5M2 and *L. buchneri* 6M1 at ratio 1:1. ^2^: IVDMD, in vitro dry matter digestibility; IVNDFD, in vitro neutral detergent fiber digestibility.

**Table 5:** Effects of new inoculant on fermentation characteristics and microbial count of corn silage ensiled for 72 d.

Item	Treatment ^1^		SEM	*P*-value
	CON	INO		
Fermentation characteristics				
pH	3.80	3.78	0.03	0.363
Ammonia-N, g/kg DM	0.60	0.50	0.05	0.537
Lactate, g/kg DM	130.0	129.0	3.01	0.864
Acetate, g/kg DM	20.9 ^b^	24.9 ^a^	1.28	0.019
Propionate, g/kg DM	ND	ND	-	
Butyrate, g/kg DM	ND	ND	-	
Lactate: acetate	6.22 ^a^	5.22 ^b^	0.314	0.002
Microbe count, log10 cfu/g				
Lactic acid bacteria	6.82	6.89	0.123	0.474
Yeast	5.89 ^a^	5.79 ^b^	0.041	0.029
Mold	ND	ND	-	

^a,b^: Mean in the same row with different superscripts differ significantly (*p* ≤ 0.05). ^1^: CON, corn silage without inoculant application; INO, corn silage inoculated with mixture of *L. brevis* 5M2 and *L. buchneri* 6M1 at ratio 1:1.
